# Triple Oxygen Isotope Measurements (Δ'^17^O) of Body Water Reflect Water Intake, Metabolism, and δ^18^O of Ingested Water in Passerines

**DOI:** 10.3389/fphys.2021.710026

**Published:** 2021-09-06

**Authors:** Pablo Sabat, Seth D. Newsome, Stephanie Pinochet, Roberto Nespolo, Juan Carlos Sanchez-Hernandez, Karin Maldonado, Alexander R. Gerson, Zachary D. Sharp, John P. Whiteman

**Affiliations:** ^1^Departamento de Ciencias Ecológicas, Facultad de Ciencias, Universidad de Chile, Santiago, Chile; ^2^Center of Applied Ecology and Sustainability (CAPES), Santiago, Chile; ^3^Department of Biology, University of New Mexico, Albuquerque, NM, United States; ^4^Instituto de Ciencias Ambientales y Evolutivas, Universidad Austral de Chile, Valdivia, Chile; ^5^Millennium Institute for Integrative Biology (iBio), Santiago, Chile; ^6^Laboratory of Ecotoxicology, University of Castilla-La Mancha, Toledo, Spain; ^7^Departamento de Ciencias, Facultad de Artes Liberales, Universidad Adolfo Ibáñez, Santiago, Chile; ^8^Biology Department, University of Massachusetts, Amherst, MA, United States; ^9^Department of Earth and Planetary Sciences, University of New Mexico, Albuquerque, NM, United States; ^10^Department of Biological Sciences, Old Dominion University, Norfolk, VA, United States

**Keywords:** birds, Δ'^17^O, evaporative water, metabolic rate, metabolic water, stable isotopes

## Abstract

Understanding physiological traits and ecological conditions that influence a species reliance on metabolic water is critical to creating accurate physiological models that can assess their ability to adapt to environmental perturbations (e.g., drought) that impact water availability. However, relatively few studies have examined variation in the sources of water animals use to maintain water balance, and even fewer have focused on the role of metabolic water. A key reason is methodological limitations. Here, we applied a new method that measures the triple oxygen isotopic composition of a single blood sample to estimate the contribution of metabolic water to the body water pool of three passerine species. This approach relies on Δ'^17^O, defined as the residual from the tight linear correlation that naturally exists between δ^17^O and δ^18^O values. Importantly, Δ'17O is relatively insensitive to key fractionation processes, such as Rayleigh distillation in the water cycle that have hindered previous isotope-based assessments of animal water balance. We evaluated the effects of changes in metabolic rate and water intake on Δ'^17^O values of captive rufous-collared sparrows (*Zonotrichia capensis*) and two invertivorous passerine species in the genus *Cinclodes* from the field. As predicted, colder acclimation temperatures induced increases in metabolic rate, decreases in water intake, and increases in the contribution of metabolic water to the body water pool of *Z. capensis*, causing a consistent change in Δ'^17^O. Measurement of Δ'^17^O also provides an estimate of the δ^18^O composition of ingested pre-formed (drinking/food) water. Estimated δ^18^O values of drinking/food water for captive *Z. capensis* were ~ −11‰, which is consistent with that of tap water in Santiago, Chile. In contrast, δ^18^O values of drinking/food water ingested by wild-caught *Cinclodes* were similar to that of seawater, which is consistent with their reliance on marine resources. Our results confirm the utility of this method for quantifying the relative contribution of metabolic versus pre-formed drinking/food water to the body water pool in birds.

## Introduction

Understanding the physiological mechanisms that species use to maintain water balance is becoming more relevant as increases in temperature and drought frequency represent significant ecological shifts that are affecting the behavior, distribution, and abundance of animals (McCarty, 2001; Albright et al., 2010; Şekercioğlu et al., 2012; IPCC, 2013; Remeš and Harmáčková, 2018). Because of their diurnal habits and high mass-specific metabolic rates, birds are particularly susceptible to increases in temperature and aridity (Riddell et al., 2021), so better understanding the environmental factors that influence their water balance is an important topic of research. Recent studies highlight that warm temperatures and reduced availability of fresh water impact key aspects of avian physiology, such as energy expenditure, body mass, thermal tolerance/conductance, and evaporative water loss (Carmi et al., 1993; Sabat et al., 2006a, 2009; Barceló et al., 2009; Gerson and Guglielmo, 2011; Smith et al., 2017; McWhorter et al., 2018). In addition to inducing physiological changes, thermal and water stress can also affect behavior, species distribution, and fitness. For instance, using a combination of physiological data, mechanistically informed models and climatic data predicted that the proportion of the ranges of the distribution of avian species with risk of lethal dehydration during heat waves will dramatically increase under future climate scenarios (Albright et al., 2017).

Most metabolic and functional processes of terrestrial animals are sensitive to water balance, where the steady state homeostatic water budget (intake = loss) assumes a constant amount of total body water:

$${\mathrm{WI}}_{D}+{\mathrm{WI}}_{\mathrm{PF}}+{\mathrm{WI}}_{M}={\mathrm{WL}}_{C}+{\mathrm{WL}}_{R}+{\mathrm{WL}}_{U}+{\mathrm{WL}}_{\mathrm{FC}}$$ (1)

where WI_D_ is drinking water; WI_PF_ is (preformed) water in food; WI_M_ is metabolic water formed in the aerobic metabolism of dietary macromolecules; WL_C_ is cutaneous loss of water thorough the skin; WL_R_ is the loss of water through respiratory surfaces; and WL_U_ and WL_FC_ are the loss of water through urine and feces, respectively. For most birds, the total evaporative water loss (TEWL), which is the sum of cutaneous and respiratory losses (WL_C_+WL_R_), accounts for between 50 and 80% of total losses depending on hydration conditions, while urine and feces (WL_U_+WL_FC_) account for only 15–30% of total water losses (Goldstein and Braun, 1986; MacMillen, 1990; Goldstein and Skadhauge, 2000). The relative contribution of WI_D_, WI_PF_, and WI_M_ to an animal’s total water budget depends on environmental conditions (e.g., temperature, humidity, and water intake), the rate and macromolecular substrate (protein, carbohydrates, and/or lipids) oxidation, and behavioral attributes (e.g., diurnal versus nocturnal activity). Ultimately, an organism’s water balance is a function of the interplay between the physical environment, the physiological and/or behavioral mechanisms for conserving water (minimizing losses), and the production of metabolic water which is directly linked to metabolic rate (Bartholomew and Cade, 1963; MacMillen, 1990; Gerson and Guglielmo, 2011; Rutkowska et al., 2016; Albright et al., 2017).

The contribution of metabolic water to the body water pool is highly variable among birds (MacMillen, 1990; Williams et al., 1993; Sabat et al., 2006a). Because TEWL accounts for the largest proportion of water lost by birds (McKechnie and Wolf, 2004), the WI_M_/TEWL ratio is especially informative: As this ratio increases toward unity, birds rely more heavily on metabolic water to maintain water balance. Importantly, the physiological traits and ecological conditions that constrain this ratio by either favoring or limiting reliance on metabolic water and thus potential independence from environmental water remain largely unknown (Bartholomew and Cade, 1963; MacMillen, 1990). For instance, what role does metabolic water production (WI_M_) play in maintaining water balance during physiological challenges related to thermoregulation? Does reliance on different water sources vary with thermoregulatory demands? Understanding these mechanisms is critical to creating accurate physiological models that can assess the ability of animals to adapt to potential threats caused by anthropogenic and natural environmental perturbations, especially increases in ambient temperature and drought frequency predicted for many regions over the next century (Walther et al., 2002; Vale and Brito, 2015; Iknayan and Beissinger, 2018). These perturbations are especially relevant for birds from the order Passeriformes because most species are diurnal and have small body masses, high body temperatures, and high mass-specific metabolic rates that make them particularly susceptible to thermal and dehydration stress (McKechnie and Wolf, 2010; Albright et al., 2017).

Several studies have shown that in comparison with their counterparts that occur in more mesic environments, birds inhabiting aridland ecosystems exhibit physiological adjustments to prevent water loss (Casotti and Braun, 2000; Williams and Tieleman, 2005; McKechnie et al., 2016; Gerson et al., 2019). However, few avian studies have examined variation in the use of potential sources of water to maintain water balance (Navarro et al., 2018; Smit et al., 2019), and even fewer studies have focused on the role of metabolic water in the body water budgets of birds (Williams, 2001; Giulivi and Ramsey, 2015). One of the primary limitations is the inability to assess the contribution of metabolic water to the body water pool without the use of injected tracers (e.g., ^2^H_2_^18^O) that require multiple captures of the same individual over short periods of time (Butler et al., 2004). Recently, Whiteman et al., (2019) proposed a new method for estimating the contribution of metabolic water to the body water pool based on the measurement of Δ'^17^O, which is the positive or negative deviation from the tight linear correlation that naturally exists between δ^17^O and δ^18^O values (Sharp et al., 2018; Whiteman et al., 2019). As shown in equation (1), body water inputs primarily include drinking water (W_D_) and food water (W_PF_), both of which are ultimately derived from meteoric water (i.e., precipitation), and metabolic water (WI_M_). Metabolic water (WI_M_) is assumed to have a Δ'^17^O value of −0.44‰ reflecting that of inhaled atmospheric oxygen (Liang et al., 2006; Wostbrock et al., 2020). In contrast, the Δ'^17^O value of W_D_ and W_PF_ is that of meteoric water, which is approximately +0.03‰ regardless of the source (Li et al., 2015; Sharp et al., 2018; Passey and Ji, 2019). Δ'^17^O values of meteoric water have this consistent value because mass-dependent fractionation associated with evaporation and condensation affects all three oxygen isotopes in a similar and predictable fashion (Sharp et al., 2018). By extension, evaporation during physiological processes (e.g., gular fluttering) should have minimal effect on Δ'^17^O values of animal body water.

A linear mixing model can be used to calculate the proportional contribution from drinking/food versus metabolic water (Whiteman et al., 2019). Because drinking/food water and metabolic water together provide 80–99% of the body water of most animals (Bryant and Froelich, 1995; Kohn, 1996), we can ignore the remaining minor contribution (1–20%) from water formed in condensation reactions from the bound oxygen in dietary macromolecules, and model bird body water (Δ'^17^O_BW_) as:

$$\Delta^{,17}O_{\mathrm{BW}}=F_{M}\times \left(-0.44\%0\right)+\left(1-F_{M}\right)\times \left(0.030\%0\right)$$ (2)

where F_M_ represents the fractional contribution to body water from metabolic water, and (1−F_M_) represents the contribution from pre-formed (drinking/food) water. Whiteman et al. (2019) showed that this equation accurately predicted relative changes in Δ’17O values of captive deer mice based on their metabolic rate and drinking water intake and that Δ’17O measurements in wild mammals appeared to reflect expected variation in relative mass-specific rates of metabolism and water intake. Continued research is required to assess additional potential predictors for this model, such as evaporation-driven variation in Δ'^17^O (as described above), and trophic enrichment in which food water is increasingly influenced by prey metabolic water for higher-trophic level consumers. Another important need is applying this simplified model (equation 2) to non-mammalian taxa.

Assuming a fixed Δ'^17^O value of 0.03‰ for meteoric water is reasonable, but the potential for variation should be noted. Regarding precipitation, patterns emerge at high and low values of δ^18^O: Δ'^17^O is closer to 0.01‰ if δ^18^O is above −10‰ and closer to 0.04‰ if δ^18^O is below −25‰ (Passey and Levin, 2021). Unique environmental conditions can alter Δ'^17^O more dramatically: For example, if ~90% of a closed water body evaporates into air with very low relative humidity, the Δ'^17^O of the remaining water may fall as low as −0.20‰ (Passey and Levin, 2021); evaporation of water from plants in very dry conditions can have a similar effect (Landais et al., 2006; Li et al., 2017). However, many environmental sources of meteoric water are not subject to the conditions required to cause such variation in meteoric water Δ'^17^O. In addition, while deviation from 0.03‰ represents important information for hydrological and geochemical studies, the mean Δ'^17^O value for meteoric water (0.03‰) is very distinct from the biological signal of metabolic water (−0.44‰).

Here, we explore Δ'17O in birds. We consider the responses of metabolic rate, TEWL, and the contribution of metabolic water to the body water pool in a widely distributed passerine, the rufous-collared sparrow (*Zonotrichia capensis*), with captive experiments of 15-day exposure to cold (15°C) followed by warm (30°C) environmental conditions. We hypothesize that birds acclimated to the cold conditions will have relatively higher resting metabolic rates (RMRs) but will consume less drinking water than when acclimated to warm conditions. We predict that these responses will yield a change in body water Δ^17^O values that reflect a net increase in the contribution of metabolic water to the body water pool during cold conditions. Unlike previous applications of oxygen isotopes that have focused exclusively on δ^18^O, our triple isotope approach is much less sensitive to evaporative ^18^O-enrichment of body water nor does it require isotopic characterization of all potential water sources. A novel contribution of our study is the application of a new analytical method that estimates the relative contributions drinking and food water (WI_D_+WI_PF_) vs. metabolic water (WI_M_) to the body water pool (Whiteman et al., 2019) based on the analysis of a single blood plasma sample. In addition to using this approach to study water balance in captive sparrows, we also report data on the contribution of metabolic water to the body water pool in two species of wild-caught songbirds in the genus *Cinclodes* (*Cinclodes oustaleti* and the *Cinclodes nigrofumosus*), a coastal group of invertivorous passerines that vary in their ability to use marine resources. Our approach combines phenomenological data collected from the field with results from laboratory experiments designed to identify the physiological mechanisms that constrain how animals respond to environmental conditions (Khaliq et al., 2014). The results improve our understanding of the physiological responses to climate change and the ultimate threats to species’ persistence.

## Materials and Methods

### Sample Collection

Our captive model species was the omnivorous rufous-collared sparrow, which is widely distributed across a range of habitats in western and southern South America (Araya et al., 2005). We captured 10 individuals using mist nets in the Quebrada de la Plata (33°31'S, 70°5'0W, ~500m elevation) in central Chile, a locality with a Mediterranean climate. Following capture, we transported birds to the laboratory for a 2-day habituation period at 22°C. The birds were maintained in individual cages (50×50×50cm) and were fed *ad libitum* with dried birdseed and water. Water was offered in inverted 100ml graduated plastic tubes that allowed birds to eat and drink in a small (~1cm^2^) container at the bottom of the tube. After the habituation period, birds were maintained at 15±0.5°C for 15days, and then at 30±0.5°C for another 15days (12:12 light:dark photoperiod). This acclimation period was long enough to ensure complete turnover of the body water pool for a 20–30g passerine (Bartholomew and Cade, 1963; Smit and Mckechnie, 2015). After each cold or warm acclimation period, we collected samples of blood (50–100μl) in the morning (09:00–11:00h) from the humeral vein using hematocrit tubes with anticoagulant (heparine). Blood samples were then centrifuged at 10,000rpm (relative centrifugal force = 9,250) for 5min during which plasma was separated from red blood cells, and then, plasma was frozen at −80°C until isotope analysis. Water intake rates were measured with the inverted graduated plastic tubes and corrected for evaporation by using control tubes located outside each experimental cage.

Wild *C. oustaleti* and *C. nigrofumosus* were collected using mist nets in the austral winter (June 2018) at Los Molles (32°14'22'S 71°30'54'W) on the central coast of Chile. Blood samples were obtained with heparinized microcapillary tubes from the humeral vein immediately after capture. Blood was centrifuged at 10,000rpm for 10min and the plasma was separated from red blood cells and stored at −80°C until isotope analysis.

### Metabolic Water Analysis

To measure Δ'^17^O, we cryogenically distilled water from 1 to 2 μl blood plasma samples in a vacuum line, then reacted it with BrF_5_ at ~300°C for 5–10min, quantitatively converting H_2_O to O_2_ and other gasses. These other gasses were removed *via* liquid nitrogen traps and the O_2_ was further purified by passing it through a zeolite molecular sieve and a gas chromatography column. O_2_ was then analyzed on a dual-inlet Thermo Scientific 253 isotope ratio mass spectrometer (Bremen, Germany) at the University of New Mexico Center for Stable Isotopes (Albuquerque, NM). The measured values of δ^17^O and δ^18^O were used to calculate Δ'^17^O (Sharp et al., 2018; Whiteman et al., 2019). At the beginning of each analytical session, we measured a local water standard (NM2: δ^18^O=−13.1‰, δ^17^O=−6.919‰) that had been calibrated against the international water standards VSMOW2 (δ^17^O=δ^18^O=0.000‰) and SLAP2 (δ^18^O=−55.5‰, δ^17^O=−29.699‰; Schoenemann et al., 2013; Sharp et al., 2016). The NM2 Δ'^17^O values associated with each measurement were then used to calculate a correction factor which we applied to the raw Δ'^17^O values of unknown samples to yield corrected values.

In addition to using Δ'^17^O values to understand reliance upon metabolic water, we used the combination of F_M_ values and δ^18^O values of body water to calculate the δ^18^O values of the combination of drinking and food water (δ^18^O_D+PF_) that birds consumed as:

$$\delta^{18}O_{\mathrm{DFW}}=\left[\delta^{18}O_{\mathrm{BW}}-\left(F_{M}\right)\times \left(\delta^{18}O_{\mathrm{Air}}\right)\right]/\left(1-F_{M}\right)$$ (3)

Here, we assumed δ^18^OAir was 19.4‰ because of fractionation that occurs during absorption of inhaled atmospheric oxygen. This fractionation depends on the efficiency of oxygen absorption (EO_2_; Epstein and Zeiri, 1988); although this efficiency was not measured in our experiment, previous research suggests that an EO_2_ of 0.4 is reasonable for small passerines (Clemens, 1988; Arens and Cooper, 2005), which in humans produces a fractionation of ~4.4‰ (Epstein and Zeiri, 1988). Although such data are lacking for our study species, applying the plausible range of fractionation values for absorbed oxygen (2–6‰) to equation 3 indicates that the resulting estimate of δ^18^O of ingested water generally changes by < 3‰, which is smaller than much of the naturally occurring variation in δ^18^O of potential water sources.

### Metabolic Rates and Total Evaporative Water Loss

At the end of the 15-day experimental period at each temperature treatment (15°C or 30°C), we measured rates of oxygen consumption (VO_2_) and TEWL for sparrows during 3–4h using standard flow-through respirometry and hygrometry methods that we have previously applied to this species (Sabat et al., 2006a). Measurements were made at ambient temperatures (T_a_) of 15.0±0.5°C and 30.0±0.5°C using an infrared O_2_-CO_2_ analyzer equipped with a hygrometer (FMS, Sable Systems^®^). All trials were conducted in metallic metabolic chambers (2000ml). Briefly, birds were placed in metabolic chambers kept at a constant temperature (15°C or 30°C) that received air free of water and CO_2_ removed *via* Drierite and CO_2_ absorbent at a flow of 750ml/min (±1%). O_2_ concentrations in the chamber were recorded during the active period between 06:00 and 18:00. Oxygen consumption was calculated according to the following equation (Lighton, 2018):

$${\mathrm{VO}}_{2}=\mathrm{FR}\times 60\times \left(F_{i}\;O_{2}-F_{e}\;O_{2}\right)/\left(1-F_{i}\;O_{2}\right)$$ (4)

where FR is the flow rate in ml min^−1^, and F_i_O_2_ and F_e_O_2_ are the fractional concentrations of inflow and outflow O_2_ in the metabolic chamber, respectively. We calculated absolute humidity (kg/m^3^) of air entering and leaving the chamber as=P/(T×Rw), where P is water vapor pressure of the air in Pascal, T is the dewpoint temperature in Kelvin, and Rw is the gas constant for water vapor (461,5 J/kgK; Lide, 2001). P was determined using the average value of the vapor pressure of the air entering the empty chamber (i.e., baseline period of 15min) before and after each experiment with a dewpoint hygrometer located in the FMS. Total evaporative water loss was calculated as TEWL=(V_e_×ρ_out_ – V_i_×ρ_in_), where TEWL is in mg/ml, ρ_in_ and ρ_out_ are the absolute humidity in kg/m^3^ of the inlet air and the outlet air, respectively, V_i_ is the flow rate of the air entering the chamber as given by the mass flow controller (750mlmin^−1^), and V_e_ is the flow of exiting air. Ve was calculated following (Williams and Tieleman, 2000) as:

$$V_{e}=V_{i}-\left[{\mathrm{VO}}_{2}\times \left(1-\mathrm{RQ}\right)\right]+V_{H2O}$$ (5)

V_in_ and VO_2_ (mlmin^−1^) are known, and we assumed a respiratory quotient (RQ) of 0.71 (Sabat et al., 2006a). Output from the H_2_O (kPa) analyzer, the oxygen analyzer (%), and the flow meter was digitalized using a Universal Interface II (Sable Systems, Nevada, United States) and recorded on a personal computer using EXPEDATA data acquisition software (Sable Systems, Nevada, United States). To estimate RMR, we averaged O_2_ concentrations of the excurrent air stream over a 20-min period after steady state was reached (Tieleman et al., 2002). We estimated the metabolic water production (WI_M_) of sparrows using the equivalence of 0.567ml H_2_O per liter O_2_ consumed (Schmidt-Nielsen, 1997). We calculated the WI_M_/TEWL ratio at different temperature treatments (15°C or 30°C). We also used equation (1) to calculate sparrow water balance, given that pre-formed water in food was negligible (WPF=0); after combining CWL and RWL into TEWL, and combining W_U_ and W_F_ into W_E_ as water losses *via* excreta, equation (1) was simplified to:

$$W_{D}+{\mathrm{WI}}_{M}=\mathrm{TEWL}+W_{E}$$ (6)

Birds were captured with permits from SAG, Chile (No. 10192/2019). All protocols were approved by the institutional Animal Care Committee of the University of Chile, following the recommendation of the ARRIVE guidelines (Kilkenny et al., 2010).

### Statistical Analysis

We evaluated the effect of thermal acclimation on RMR, TEWL, and water intake using a generalized linear mixed model (GLMM) with body mass as a covariate, acclimation temperature (15°C and 30°C) as fixed factors, and individual identity as a random factor to control for repeated measures. Assumptions of normality and heteroscedasticity in residuals were examined with Q–Q plots and a plot of residuals against fitted values, respectively (Zuur et al., 2009). Dependent variables and covariates were natural log transformed for data normalization. Body mass and isotope values (Δ^17^O, δ^18^O, and δ^17^O) were compared between warm (30°C) and cold (15°C) treatments using non-parametric two-sample paired t-tests. The statistical analyses were performed in nlme package (Pinheiro et al., 2013) using the R platform (v4.0.3; R Development Core Team, 2013).

## Results

### Physiological Data of Captive Sparrows

**Figure 1:**
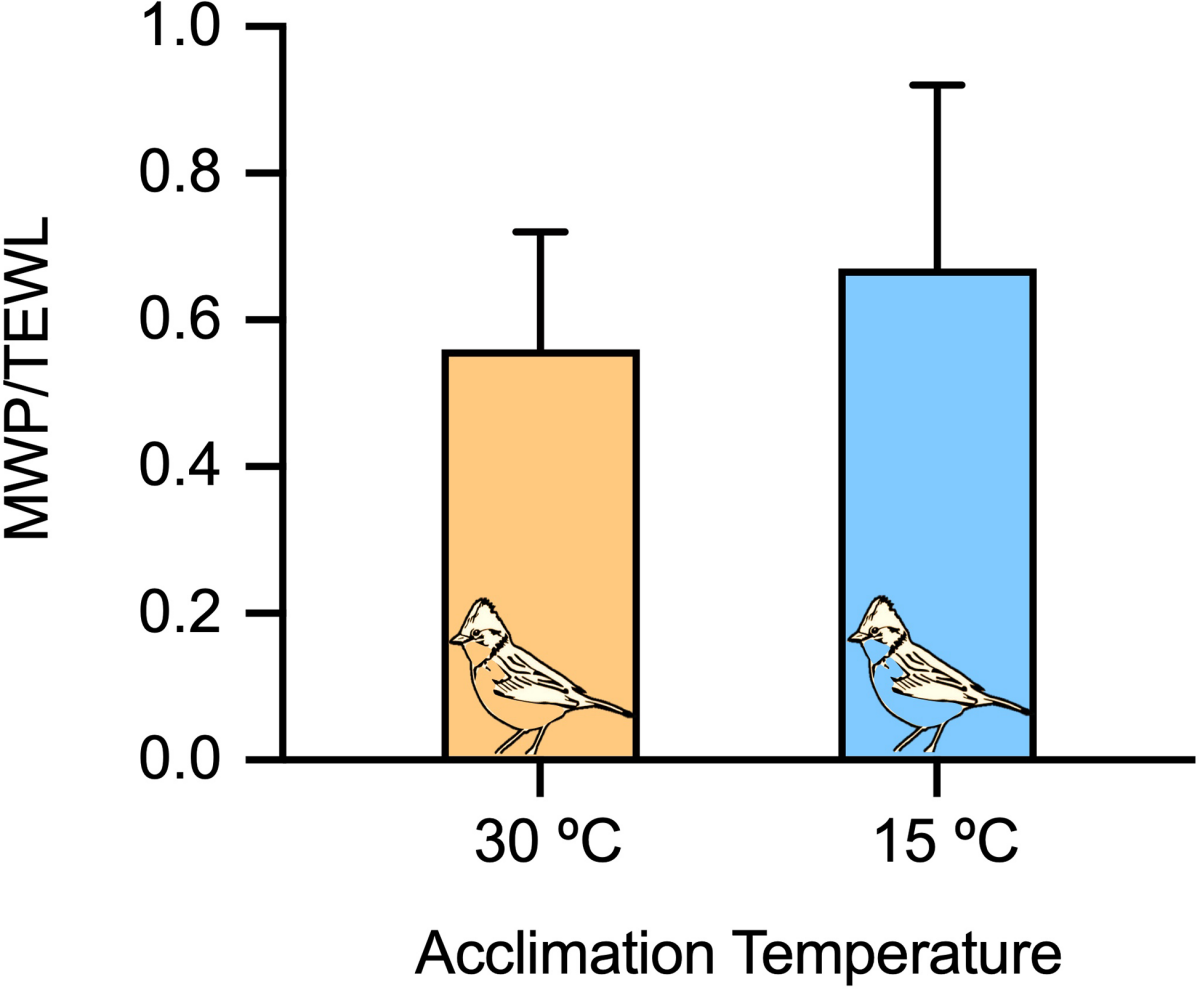
The ratio between metabolic water production estimated from RMRs and total evaporative water loss in *Z. capensis* at 15°C and 30°C ambient temperature. Error bars in each panel represent *SD*.

Sparrows acclimated at 15°C exhibited higher RMR (93.2±15.2ml O_2_ h^−1^) and lower daily water intake (0.19±0.05ml H_2_Oh^−1^) in comparison with when they were acclimated at 30°C (RMR: 70.8±12.2ml O_2_ h^−1^ and daily water intake: 0.26±0.08ml H_2_Oh^−1^); however, there was no difference in TEWL between temperature treatments (Table 1). In addition, the WI_M_/TEWL ratio, converted to percentage, decreased significantly with increasing T_a_ (Figure 1), ranging from ~65% at 15°C to ~55% at 30°C. Using W_IM_ and W_D_ and the equation (6), metabolic water represented 22.8±4.2% and 14.0±5.6% of the total body water pool (i.e., W_IM_/(W_IM_+W_D_) at 15°C and 30°C respectively, while TEWL represented 80% of the total water loss at 15°C and 67% of the total water loss at 30°C.

**Table 1:** Results of generalized linear mixed model testing the effect of 15days of thermal acclimation to 15°C (cold) and 30°C (warm) on resting metabolic rate (RMR), total evaporative water loss (TEWL), and water intake in *Zonotrichia capensis*.

Predictors	Coeff. Estimate	95% CI	*df*	*t*	Value of *p*	*R*^2^ marginal/conditional
**RMR (mlO_2_ h^−1^)**						0.58/0.58
Intercept	0.94	(−1.91–3.79)	9	0.75	0.47	
Mass	1.18	(0.22–2.13)	8	2.84	**0.02**	
Temperature (30°)	−2.25	(−0.40 to −0.10)	8	−3.85	**0.005**	
**TEWL (mgH_2_O h^−1^)**						0.10/0.25
Intercept	2.52	(−2.95–8.00)	9	1.04	0.32	
Mass	0.62	(−1.21–2.45)	8	0.78	0.45	
Temperature (30°)	−0.12	(−0.37–0.13)	8	−1.15	0.28	
**Water Intake (mlH_2_O)**						0.25/0.66
Intercept	−2.45	(−8.96–4.06)	9	−0.85	0.41	
Mass	0.24	(−1.94–2.42)	8	0.26	0.80	
Temperature (30°)	0.34	(0.13–0.55)	8	3.67	**0.006**	

Numbers in bold indicate statistical significance (*p*<0.05)

### Oxygen Isotopes

**Figure 2:**
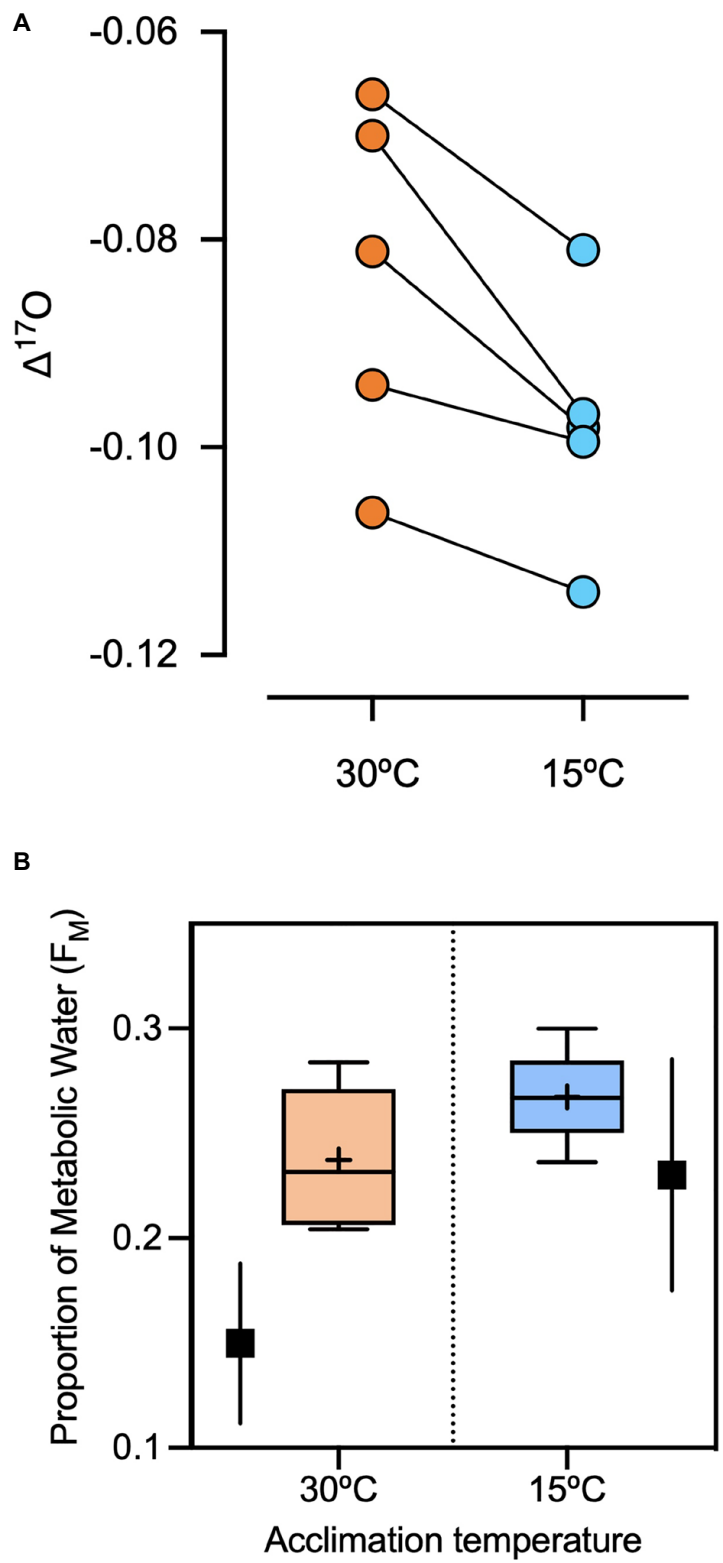
**(A)** The effects of changes in metabolic rate and water intake on Δ'^17^O values of five *Z. capensis*. Individuals were kept in standard housing conditions at 30°C, then exposed to 15°C for 15days. The cooler temperature, outside of their thermal neutral zone, induced increases in RMR by a mean (±*SD*) of 31%±12% (data not shown) and decreases in mean drinking water intake (−17%±28%). These changes should have increased the contribution of metabolic water to the body water pool. Accordingly, the Δ'^17^O values of plasma samples significantly declined from 30°C to 15°C, reflecting the greater contribution of metabolic water (Δ'^17^O=−0.44‰) and smaller contribution of drinking water (Δ'^17^O=0.03‰). **(B)** The mean proportion of metabolic water in the body water pool water in captive *Z. capensis* estimated from Δ'^17^O of plasma at 15°C and 30°C ambient temperature. Values from physiological experiments (W_IM_/(W_IM_+W_D_) are included in filled black squares and its error for comparison.

**Figure 3:**
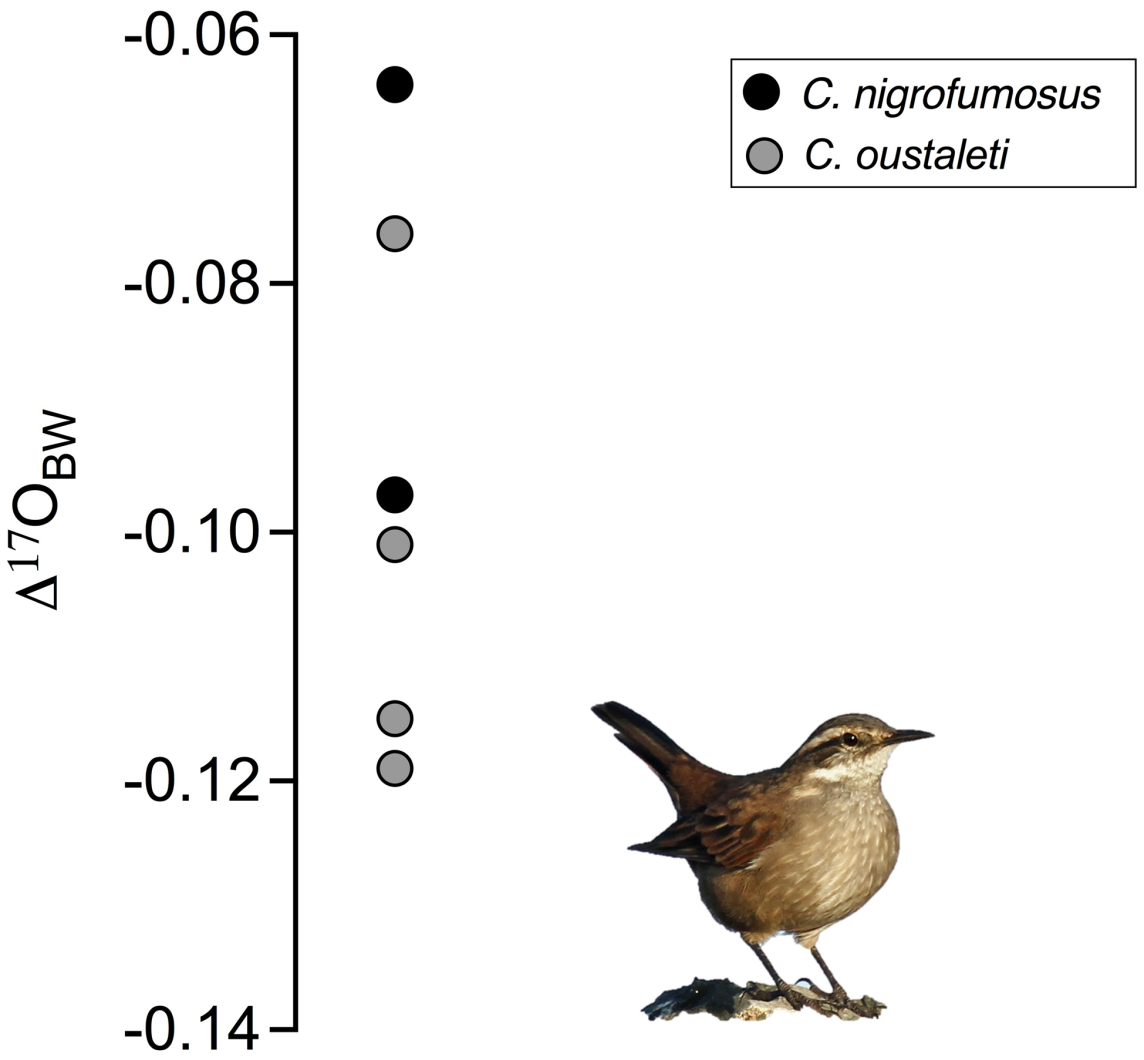
Δ'^17^O values for six *Cinclodes* individuals sampled at Los Molles, a coastal locality in central Chile characterized by moderate temperatures and low precipitation.

Δ'^17^O values were lower for captive sparrows acclimated to 15°C than 30°C (Wilcoxon Sign test=−7.5; *p*=0.03, Figure 2A). Δ'^17^O-based estimates (*via* equation 2) of the proportion of metabolic water in the total body water pool in captive sparrows were 27.2 and 24.1% at 15°C and 30°C, respectively (Figure 2B). Wild *Cinclodes* Δ'^17^O values vary both within and among species (Figure 3), and Δ'^17^O-based estimates of the metabolic water contribution to the body water pool ranged from 19.6 to 31.0%. Mean (±SD) metabolic water contributions for *C. nigrofumosus* and *C. oustaleti* were 23.0±4.8% and 27.7±4.0%, respectively. Intriguingly, two of the three highest measured Δ'^17^O values were sourced from *C. nigrofumosus*, suggesting a greater intake of pre-formed drinking/food water than for *C. oustaleti*. This inference is consistent with the general hypothesis of reduced dependence on WI_M_ for larger body-sized individuals across birds and mammals (Whiteman et al., 2019), because *C. nigrofumosus* (70–80g) is more than twice the body mass of *C. oustaleti* (23–28g; Sabat et al., 2006a). The mean (±*SD*) estimated δ^18^O value of the combined pre-formed drinking/food water ingested by captive sparrows was ~ −11±3‰, which is within the range for tap water and groundwater in the Santiago Basin of central Chile (−15 to −11‰; Iriarte et al., 2004). In contrast, the pre-formed drinking/food water ingested by wild *Cinclodes* was estimated to have mean δ^18^O (±*SD*) values near seawater (0‰; LeGrande and Schmidt, 2006): 0.2±8.3‰ for *C. nigrofumosus* and −1.4±0.9‰ for *C. oustaleti*.

## Discussion

Metabolic water production (WI_M_) alone is typically insufficient to meet the water requirements of most vertebrates (McNab, 2002), although some species adapted to arid environments or routinely experience long periods of time without food or drinking water can survive solely on metabolic water under certain conditions (Bartholomew and Cade, 1963; MacMillen and Hinds, 1983; Ostrowski et al., 2002). Several factors have been suggested to influence the importance of WI_M_ to the water budget of birds, such as the nature of oxidized substrates and environmental temperature. The current study aimed to experimentally assess the effects of changes in metabolic rate and water intake on Δ'^17^O values of captive house sparrows, kept in standard housing conditions at 30°C or 15°C for 15days. As predicted, sparrows acclimated to cooler temperatures increased their RMR by ~31% while decreasing their drinking water intake by ~27% (Table 1). This elevation in metabolic rate suggests that an ambient temperature of 15°C is below their lower critical temperature (Maldonado et al., 2009). Accordingly, plasma Δ'^17^O values were lower in individuals housed at 15°C than at 30°C, reflecting the larger contribution of metabolic water relative to drinking/food water to the body water pool (Figure 2). These results support the predictions of a model of the relationship between the WI_M_:TEWL ratio and ambient temperature in granivorous birds (MacMillen, 1990). In this model, the ratio of WI_M_/TEWL increases when temperature declines below the T_LC_, as a result of increased WI_M_ and dampened TEWL *via* water-recovery adaptations, such as desaturation of exhaled air into nasal surfaces (MacMillen, 1990). In a similar controlled experiment on mammals, Δ'^17^O values of plasma from deer mice declined in response to elevated metabolic rate when animals were housed at 5°C rather than 25°C (Whiteman et al., 2019). The magnitude of the decline in Δ'^17^O (0.01–0.03 ‰) in deer mice was similar to that observed here in captive sparrows. Our results confirm that WI_M_ in small passerines increases with thermoregulatory demands and highlights the utility of this method for estimating water balance in laboratory conditions.

In a formative study, it was observed that the theoretical production of metabolic water can approach the rate of evaporative water loss in birds with body masses > 60g, creating the opportunity for these larger birds to be “water-independent” and possibly rely solely on WI_M_ (Bartholomew and Cade, 1963). However, MacMillen (1990) suggested that smaller birds (e.g., < 20g) can also attain favorable states of water balance (WI_M_>TEWL) if they are below the lower critical temperature (~25°C) when experiencing water deprivation. For the captive sparrows (~20g) in our study, we calculated a mean WI_M_/TEWL of ~61% (Figure 1), which suggests that sparrows are not capable of relying solely on metabolic water production to maintain water balance, even in conditions that yield increases in metabolic rate (i.e., colder temperatures). Note that WI_M_/TEWL was 67 and 56% for cold- and warm-acclimated birds, respectively. Nevertheless, our estimates of TEWL come from an experimental setup that controlled the humidity inside the metabolic chambers near zero, which likely does not occur often in nature. In some birds, TEWL appears to vary as a function of absolute humidity (mgH_2_O/m^3^) across a range of environmental temperatures (Powers 1992, Gerson et al., 2014). By using data at comparable temperatures from the literature and climate data available from a local weather station,^1^ we calculated that TEWL could be reduced on average by up to 20% at 30°C and 40% at 20°C, which would result in a WIM/TEWL of 76% at 30°C and up to 100% at 15°C.

The traditional approach to measuring WI_M_ is to assume a constant equivalence of water production based on oxygen consumption (Schmidt-Nielsen, 1997). Using this approach, we found that WI_M_ was 23% of the total water intake (i.e., WI_M_+W_D_) at 15°C but decreased to 14% at 30°C. These values are slightly lower to the percent contribution of metabolic water to the total body water pool that we estimated from Δ'^17^O data alone, which were 27.5% for cold-acclimated birds and 24.5% for warm-acclimated birds. The similarity in estimates of WI_M_ using the two approaches is notable because the Δ'^17^O estimates were based on collection of a single sample and did not rely on any measurements of water intake or loss, highlighting the potential accuracy of this new method (Whiteman et al., 2019). The discrepancy in estimates of WI_M_, which is larger for warm-acclimated sparrows (14.0% vs. 24.5%), may be due to at least two non-exclusive alternatives. First, the calculation for the amount of water consumed per day was obtained under acclimatization conditions when birds were in larger cages that allowed for movement and flight, conditions that yield higher rates of energy consumption than in the more confined conditions when RMR was measured. In addition, the traditional approach of using an equivalency between oxygen consumption and WI_M_ (i.e., 0.567ml H_2_O per liter O_2_ consumed) does not distinguish between (1) H_2_O that was produced by condensation reactions that occur during the oxidation of food that contains oxygen bound in macromolecules (e.g., protein or lipids) and (2) H_2_O that was produced by complex IV of the electron transport chain, and which therefore only contains inhaled atmospheric oxygen (Morrison, 1953). In contrast, the Δ'^17^O approach only estimates the contribution of the latter mitochondrial source of H_2_O. It should also be noted that body water exchanges oxygen atoms with dissolved CO_2_ in the blood *via* the bicarbonate buffer system. This CO_2_ is from metabolic decarboxylation (e.g., reactions that occur within the citric acid cycle) and contains oxygen bound in macromolecules (e.g., glucose) as well as phosphate groups (e.g., added by glucokinase). We expect that the influence of dissolved CO_2_ on body water Δ'^17^O is small, because the isotopic fractionation associated with loss of exhaled CO_2_ (Speakman and Racey, 1987; Haggarty et al., 1988) is mass-dependent, and because phosphate groups are likely in isotopic equilibrium with body water (Li et al., 2016). In general, more precise studies of water balance that considers the loss of water through urine and measurements of metabolic rate during longer periods in acclimatization conditions are necessary to establish the precision of the Δ'^17^O-based method.

The contribution of metabolic water to the body water pool is highly variable among birds, ranging from < 10% in some hummingbirds, ~14% in desert-adapted ostriches, and up to 80% in some passerines (MacMillen, 1990; Williams et al., 1993). For example, captive zebra finches (*Taeniopygia guttata*) with *ad libitum* access to drinking water produced only 1ml metabolic H_2_O per ~1.5–1.8ml of evaporative water lost at temperatures between 15 and 25°C, showing that without drinking water, the birds would have been in negative water balance. However, when birds were dehydrated for 30days, their TEWL declined and WI_M_/TEWL increased to one (Cade et al., 1965). The influence of water availability on the relative importance of WI_M_ to total water pool in birds is in agreement with our previous work on small mammals. For example, captive mice (*Mus musculus*) that were provided drinking water *ad libitum* had smaller contributions of metabolic water to their body water than did wild desert-adapted small mammals (*Peromyscus leucopus*) of similar body mass (Whiteman et al., 2019). Overall, these results suggest that wild animals lacking *ad libitum* access to water responded by relying more on metabolic water than their captive counterparts.

Birds also have substantial flexibility in their sources of water intake. In wild zebra finches, WI_M_ calculated on the basis of field metabolic rate was lower during hot versus cool periods. As a consequence, WI_M_ fulfilled 20% of water requirements during hot days and 32% on cold days (Cooper et al., 2019). Because the total water turnover (ml/day) did not vary substantially with environmental temperature, the change in the contribution of metabolic water to the total body water pool (i.e., *F_M_*) is likely due to changes in metabolic rates. A question that remains unresolved is whether birds, especially species living in arid seasonal environments, modify their metabolic rate strictly for the purpose of WI_M_. Lastly, another important variable that influences WI_M_ is the type of oxidative substrate used for aerobic metabolism. For instance, Zebra finches predictably lose body mass when fasting; however, when they are simultaneously water deprived, they lose substantially more body fat than lean (protein-rich) tissue (Rutkowska et al., 2016). The catabolism of body fat can potentially substantially increase the yield of metabolic water because fat is far more energy dense than other macromolecules oxidized for energy. In contrast, house sparrows (*Passer domesticus*) accelerate protein catabolism during acute dehydration (Gerson and Guglielmo, 2011), presumably to liberate pre-formed water molecules bound in proteinaceous (muscle) tissue (Giulivi and Ramsey, 2015).

We compared the Δ'^17^O-based estimates of the fractional contribution to body water from metabolic water (*F_M_*) in captive sparrows with similar estimates for arid- and mesic-adapted wild birds based on allometric equations for water influx rate and field metabolic rate (Williams et al., 1993). We assumed that WI_M_ from oxidized substrates is 0.027 mlH_2_O/KJ based on oxidation of carbohydrates; note that this calculation yields the same result for WI_M_ as the equivalence method (0.567ml H_2_O per liter O_2_) mentioned above (Morrison, 1953). We found that on average, the warm- and cold-acclimated sparrows in our study had *F_M_* values that were~29% and~26% higher than the values expected for arid- and mesic-adapted birds of a similar size. Estimates of *F_M_* in wild *C. nigrofumosus* were~23% or~19% higher than expected values for similar-sized birds inhabiting an arid or mesic environment, respectively. Note that Δ'^17^O-based estimates of *F_M_* for captive sparrows and wild *C. nigrofumosus* are within the 95% confidence interval of those reported for arid- and mesic-adapted birds of similar size. In contrast, Δ'^17^O-based estimates of *F_M_* for *C. oustaleti* were significantly higher by ~48% or~42% than predicted for birds from arid and mesic environments, respectively. We hypothesize that observed differences in *F_M_* based on Δ'^17^O versus allometric proxies for *C. oustaleti* may be related to the high energetic and osmoregulatory costs of migrating between coastal (winter) and high elevation (summer) habitats, which may also be why this species has a relatively high BMR in comparison with other *Cinclodes* (Tapia-Monsalve et al., 2018). Overall, Δ'^17^O data revealed that the contribution of metabolic water to body water in captive sparrows and wild *Cinclodes* was similar to or within the same order of magnitude as for other free-ranging birds based on logarithmic allometric relationships. This finding emphasizes the validity of our method to the study of wild birds in natural ecosystems.

**Figure 4:**
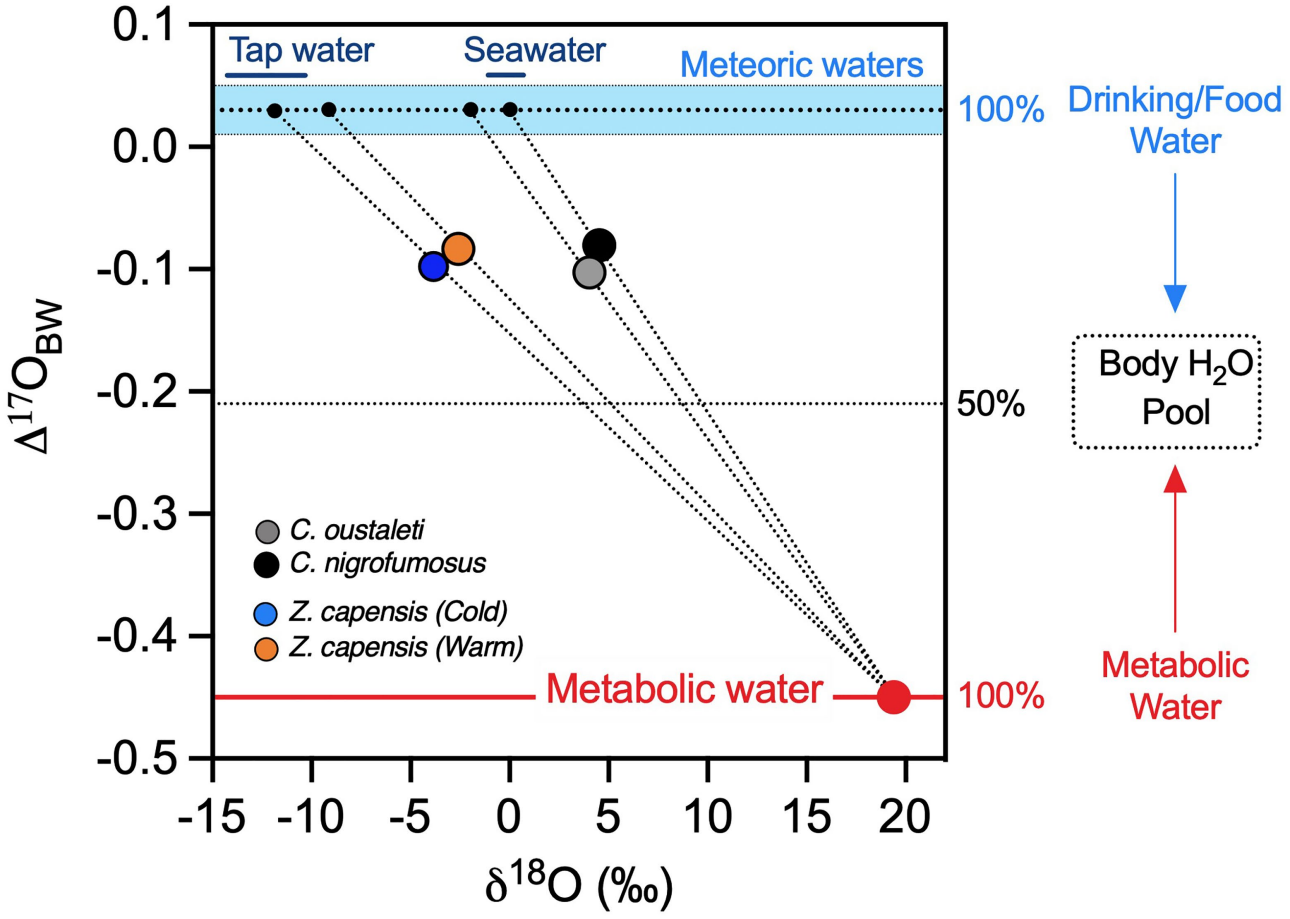
Measured mean Δ'^17^O values (y-axis) and δ^18^O values (x-axis) of body water used to estimate the δ^18^O value of ingested drinking/food water of captive *Z. capensis* and wild *Cinclodes*. Captive *Z. capensis* was predicted to have ingested drinking/food water with a δ^18^O value of −12‰, which is consistent with tap water in Santiago, Chile. *Cinclodes* were predicted to have ingested drinking/food water with a δ^18^O value of ~0‰, similar to seawater and consistent with their reliance on marine resources. Ranges of δ^18^O values for tap water and seawater are shown by solid bars at the top of the range for meteoric waters (sky-blue dashed area). The dotted lines represent the solutions for equation 2 for each group of birds.

As expected, our regression approach based on oxygen isotope analysis shows that captive sparrows consumed drinking/food water that had a δ^18^O value of −11‰, consistent with local tap water in Santiago, Chile (Figure 4.). Likewise, isotope data show that wild *Cinclodes* consumed pre-formed drinking/food water with δ^18^O values of ~0‰, which is consistent with the oxygen isotope composition of seawater (Figure 4). Intriguingly, one *C. nigrofumosus* individual ingested water with an anomalously positive estimated δ^18^O value of 6.3‰ (Supplementary Table S1), notably higher than seawater. This enrichment could result from abiotic evaporation occurring in the environment prior to ingestion and/or physiologically mediated evaporative enrichment occurring within the organism. The first explanation suggests seawater ingested as pre-formed drinking/food water in the arid intertidal habitats where *C. nigrofumosus* forages in central and northern Chile may be ^18^O-enriched. Alternatively, the relatively high δ^18^O_DFW_ value observed for this species may result from isotopic fractionation that occurs during evaporation of body water, which largely depends on environmental temperature and humidity (Kohn, 1996). While *C. nigrofumosus* in this study was sampled in arid central Chile where evaporative ^18^O-enrichment of body water could be a factor, this species inhabits humid coastal intertidal ecosystems and has ample access to drinking water in the form of seawater (Sabat et al., 2006a). In contrast to patterns for wild *Cinclodes*, we observed less within-treatment variation in estimates of δ^18^O_DFW_ for captive sparrows (Supplementary Table S1), which is expected because (1) their drinking water was sourced from a municipal groundwater-derived aquifer with a relatively constant δ^18^O value and (2) they were subjected to less variation in temperature and humidity in comparison with wild *Cinclodes* species (Sabat and del Rio, 2002; Sabat et al., 2006a). To better understand the ecological (e.g., habitat use and/or diet composition) and environmental (e.g., temperature and/or humidity) factors that influence water budgets in birds will require more experiments that assess the effect of physiologically mediated water conservation strategies on the oxygen isotope composition of body water, and additional sampling of birds from a range of environments that span temperature and humidity gradients.

Finally, apparent differences in δ^18^O of pre-formed drinking/food water between *Cinclodes* species may be related to inter-specific variation in the ability to cope with saline-rich prey and drinking water sources (Sabat et al., 2006b; Tapia-Monsalve et al., 2018). For example, the high salinity tolerance of *C. nigrofumosus* (Sabat et al., 2006b), especially at the northern margin of its distribution, may allow this species to rely less on metabolic water because of the ability to obtain and process an abundant source of drinking/food water sourced from the ocean. The ecological conditions that either favor or limit the ability of birds to depend on metabolic water remain largely unknown, while the combined effect of temperature and humidity on a bird’s water budget is poorly understood, especially in free-ranging individuals. We predict that a species dependence on metabolic water vs. pre-formed drinking/food water along environmental gradients will depend on the interaction between the ability of different populations/species to retain water and dissipate heat, coupled with the availability of pre-formed water as well as environmental temperature and humidity. For example, the study of bird species and/or populations that differ in how they evaporatively cool their bodies *via* panting, hyperthermia, or cutaneous evaporation that ultimately influences water loss rates (Gerson et al., 2014) represents a unique opportunity to determine which environmental and physiological variables modulate an animal’s use of metabolic versus pre-formed water to maintain water balance.

Overall, our study revealed that the contribution of metabolic water to the total body water pool increased with metabolic rate, consistent with a recent report in small mammals that used a similar Δ'^17^O-based approach (Whiteman et al., 2019). Although these increases may not seem very significant in magnitude, they could account for natural changes in the energy expenditure of animals in the field. More precise studies of water balance that considers all potential sources of water losses during longer periods in acclimatization are necessary to establish the precision of the Δ'^17^O-based method used in this study. Δ'^17^O-based estimates of *F_M_* in captive sparrows and wild *C. nigrofumosus* were similar to those for free-ranging birds based on independent allometric relationships, while estimates for *C. oustaleti* based on oxygen isotopes were higher than expected, but in the same order of magnitude as predictions based on logarithmic allometric relationships, emphasizing the validity of our method to better understand water balance in wild birds.

## Data Availability Statement

The raw data supporting the conclusions of this article will be made available by the authors, without undue reservation.

## Ethics Statement

The animal study was reviewed and all protocols were approved by the institutional Animal Care Committee of the University of Chile (CICUA), and National Research and Development Agency (ANID).

## Author Contributions

PS, SN, and JW designed the research. SP, PS, ZS, and JW performed the research. SP, AG, KM, and PS analyzed the data. PS, SN, RN, JS-H, KM, and JW wrote the paper. All authors contributed to the article and approved the submitted version.

## Conflict of Interest

The authors declare that the research was conducted in the absence of any commercial or financial relationships that could be construed as a potential conflict of interest.

## Publisher’s Note

All claims expressed in this article are solely those of the authors and do not necessarily represent those of their affiliated organizations, or those of the publisher, the editors and the reviewers. Any product that may be evaluated in this article, or claim that may be made by its manufacturer, is not guaranteed or endorsed by the publisher.

## Footnotes

^1^ www.meteored.cl

## References

[ref1] Albright T. P. Mutiibwa D. Gerson A. R. Smith E. K. Talbot W. A. O’Neill J. J.. (2017). Mapping evaporative water loss in desert passerines reveals an expanding threat of lethal dehydration. Proc. Natl. Acad. Sci. U. S. A. 114, 2283–2288. 10.1073/pnas.1613625114, PMID: 28193891 PMC5338552

[ref2] Albright T. P. Pidgeon A. M. Rittenhouse C. D. Clayton M. K. Wardlow B. D. Flather C. H.. (2010). Combined effects of heat waves and droughts on avian communities across the conterminous United States. Ecosphere 1, 1–22. 10.1890/ES10-00057.1

[ref3] Araya B. Millie G. BERNAL M. (2005). Guía de campo de las aves de Chile. Editor. Univ. Santiago, 406.

[ref4] Arens J. R. Cooper S. J. (2005). Seasonal and diurnal variation in metabolism and ventilation in house sparrows. Condor 107, 433–444. 10.1093/condor/107.2.433

[ref5] Barceló G. Salinas J. Cavieres G. Canals M. Sabat P. (2009). Thermal history can affect the short-term thermal acclimation of basal metabolic rate in the passerine *Zonotrichia capensis*. J. Therm. Biol. 34, 415–419. 10.1016/j.jtherbio.2009.06.008

[ref6] Bartholomew G. A. Cade T. J. (1963). The water economy of land birds. Auk 80, 504–539. 10.2307/4082856

[ref7] Bryant D. J. Froelich P. N. (1995). A model of oxygen isotope fractionation in body water of large mammals. Geochim. Cosmochim. Acta 59, 4523–4537. 10.1016/0016-7037(95)00250-4

[ref005] Butler P. J. Green J. A. Boyd I. L. Speakman J. R. (2004). Measuring metabolic rate in the field: the pros and cons of the doubly labelled water and heart rate methods. Funct. Ecol. 18, 168–183. 10.1086/physzool.38.1.30152342

[ref8] Cade T. J. Tobin C. A. Gold A. (1965). Water economy and metabolism of two Estrildine finches. Physiol. Zool. 38, 9–33. 10.1086/physzool.38.1.30152342

[ref9] Carmi N. Pinshow B. Horowitz M. Bernstein M. H. (1993). Birds conserve plasma volume during thermal and flight-incurred dehydration. Physiol. Zool. 66, 829–846. 10.1086/physzool.66.5.30163826

[ref10] Casotti G. Braun E. J. (2000). Renal anatomy in sparrows from different environments. J. Morphol. 243, 283–291. 10.1002/(SICI)1097-4687(200003)243:3<283::AID-JMOR5>3.0.CO;2-B, PMID: 10681473

[ref11] Clemens D. T. (1988). Ventilation and oxygen consumption in rosy finches and house finches at sea level and high altitude. J. Comp. Physiol. B 158, 57–66. 10.1007/BF00692729

[ref12] Cooper C. E. Withers P. C. Hurley L. L. Griffith S. C. (2019). The field metabolic rate, water turnover, and feeding and drinking behavior of a small Avian Desert Granivore During a summer heatwave. Front. Physiol. 10:1405. 10.3389/fphys.2019.01405, PMID: 31824330 PMC6879461

[ref13] Epstein S. Zeiri L. (1988). Oxygen and carbon isotopic compositions of gases respired by humans. Proc. Natl. Acad. Sci. 85, 1727–1731. 10.1073/pnas.85.6.1727, PMID: 3162303 PMC279851

[ref14] Gerson A. R. Guglielmo C. G. (2011). House sparrows (Passer domesticus) increase protein catabolism in response to water restriction. Am. J. Phys. Regul. Integr. Comp. Phys. 300, R925–R930. 10.1152/ajpregu.00701.2010, PMID: 21248307

[ref15] Gerson A. R. McKechnie A. E. Smit B. Whitfield M. C. Smith E. K. Talbot W. A.. (2019). The functional significance of facultative hyperthermia varies with body size and phylogeny in birds. Funct. Ecol. 33, 597–607. 10.1111/1365-2435.13274

[ref16] Gerson A. R. Smith E. K. Smit B. McKechnie A. E. Wolf B. O. (2014). The impact of humidity on evaporative cooling in Small Desert birds exposed to high air temperatures. Physiol. Biochem. Zool. 87, 782–795. 10.1086/678956, PMID: 25461643

[ref17] Giulivi C. Ramsey J. (2015). On fuel choice and water balance during migratory bird flights. Int. Biol. Rev. 2015:58. 10.18103/ibr.v0i1.58, PMID: 28203650 PMC5304341

[ref18] Goldstein D. L. Braun E. J. (1986). Lower intestinal modification of ureteral urine in hydrated house sparrows. Am. J. Phys. Regul. Integr. Comp. Phys. 250, R89–R95. 10.1152/ajpregu.1986.250.1.R89, PMID: 3942257

[ref19] Goldstein D. L. Skadhauge E. (2000). “Renal and Extrarenal regulation of body fluid composition,” in Sturkie’s Avian Physiology. ed. Whittow G. C. (Elsevier), 265–297.

[ref20] Haggarty P. McGaw P. A. Franklin M. F. (1988). Measurement of fractionated water loss and CO2 production using triply labelled water. J. Theor. Biol. 134, 291–308. 10.1016/S0022-5193(88)80060-2, PMID: 3151080

[ref21] Iknayan K. J. Beissinger S. R. (2018). Collapse of a desert bird community over the past century driven by climate change. Proc. Natl. Acad. Sci. U. S. A. 115, 8597–8602. 10.1073/pnas.1805123115, PMID: 30082401 PMC6112692

[ref22] IPCC (2013). Intergovernmental Panel on Climate Change Working Group I. Climate Change 2013: The Physical Science Basis. Long-term Climate Change: Projections, Commitments and Irreversibility. *Cambridge Univ. Press. New York*.

[ref23] Iriarte S. Tore C. Pardo M. Aguirre E. Aravena E. R. (2004). Use of environmental isotopes to evaluate natural and antropic sources of groundwater in an area with multiple land uses, Santiago norte basin, Chile. Isot. Hydrol. Integr. Water Resour. Manage., 165–167.

[ref24] Khaliq I. Hof C. Prinzinger R. Böhning-Gaese K. Pfenninger M. (2014). Global variation in thermal tolerances and vulnerability of endotherms to climate change. Proc. R. Soc. B Biol. Sci. 281:20141097. 10.1098/rspb.2014.1097, PMID: 25009066 PMC4100521

[ref25] Kilkenny C. Browne W. J. Cuthill I. C. Emerson M. Altman D. G. (2010). Improving bioscience research reporting: the ARRIVE guidelines for reporting animal research. PLoS Biol. 8:e1000412. 10.1371/journal.pbio.1000412, PMID: 20613859 PMC2893951

[ref26] Kohn M. J. (1996). Predicting animal δ18O: accounting for diet and physiological adaptation. Geochim. Cosmochim. Acta 60, 4811–4829. 10.1016/S0016-7037(96)00240-2

[ref27] Landais A. Barkan E. Yakir D. Luz B. (2006). The triple isotopic composition of oxygen in leaf water. Geochim. Cosmochim. Acta 70, 4105–4115. 10.1016/j.gca.2006.06.1545

[ref28] LeGrande A. N. Schmidt G. A. (2006). Global gridded data set of the oxygen isotopic composition in seawater. Geophys. Res. Lett. 33:L12604. 10.1029/2006GL026011

[ref29] Li S. Levin N. E. Chesson L. A. (2015). Continental scale variation in 17O-excess of meteoric waters in the United States. Geochim. Cosmochim. Acta 164, 110–126. 10.1016/j.gca.2015.04.047

[ref30] Li S. Levin N. E. Soderberg K. Dennis K. J. Caylor K. K. (2017). Triple oxygen isotope composition of leaf waters in Mpala, Central Kenya. Earth Planet. Sci. Lett. 468, 38–50. 10.1016/j.epsl.2017.02.015

[ref31] Li H. Yu C. Wang F. Chang S. J. Yao J. Blake R. E. (2016). Probing the metabolic water contribution to intracellular water using oxygen isotope ratios of PO4. Proc. Natl. Acad. Sci. U. S. A. 113, 5862–5867. 10.1073/pnas.1521038113, PMID: 27170190 PMC4889363

[ref32] Liang M.-C. Irion F. W. Weibel J. D. Miller C. E. Blake G. A. Yung Y. L. (2006). Isotopic composition of stratospheric ozone. J. Geophys. Res. 111:D02302. 10.1029/2005JD006342

[ref33] Lide D. R. (2001). CRC Handbook of Chemistry and Physics. Boca Raton, FL: CRC press.

[ref34] Lighton J. R. B. (2018). “Flow-through Respirometry: The equations,” in Measuring Metabolic Rates (Oxford: Oxford University Press), 94–100.

[ref35] MacMillen R. E. (1990). Water economy of Granivorous birds: a predictive model. Condor 92, 379–392. 10.2307/1368235

[ref36] MacMillen R. E. Hinds D. S. (1983). Water regulatory efficiency in Heteromyid rodents: a model and its application. Ecology 64, 152–164. 10.2307/1937337

[ref37] Maldonado K. E. Cavieres G. Veloso C. Canals M. Sabat P. (2009). Physiological responses in rufous-collared sparrows to thermal acclimation and seasonal acclimatization. J. Comp. Physiol. 179, 335–343. 10.1007/s00360-008-0317-1, PMID: 19011873

[ref38] McCarty J. P. (2001). Ecological consequences of recent climate change. Conserv. Biol. 15, 320–331. 10.1046/j.1523-1739.2001.015002320.x

[ref39] McKechnie A. E. Smit B. Whitfield M. C. Noakes M. J. Talbot W. A. Garcia M.. (2016). Avian thermoregulation in the heat: evaporative cooling capacity in an archetypal desert specialist, Burchell’s sandgrouse (Pterocles burchelli). J. Exp. Biol. 219, 2137–2144. 10.1242/jeb.139733, PMID: 27207634

[ref40] McKechnie A. E. Wolf B. O. (2004). The allometry of avian basal metabolic rate: good predictions need good data. Physiol. Biochem. Zool. 77, 502–521. 10.1086/383511, PMID: 15286923

[ref41] McKechnie A. E. Wolf B. O. (2010). Climate change increases the likelihood of catastrophic avian mortality events during extreme heat waves. Biol. Lett. 6, 253–256. 10.1098/rsbl.2009.0702, PMID: 19793742 PMC2865035

[ref42] McNab B. K. (2002). Short-term energy conservation in endotherms in relation to body mass, habits, and environment. J. Therm. Biol. 27, 459–466. 10.1016/S0306-4565(02)00016-5

[ref43] McWhorter T. J. Gerson A. R. Talbot W. A. Smith E. K. McKechnie A. E. Wolf B. O. (2018). Avian thermoregulation in the heat: evaporative cooling capacity and thermal tolerance in two Australian parrots. J. Exp. Biol. 221:jeb168930. 10.1242/jeb.168930, PMID: 29440360

[ref44] Morrison S. D. (1953). A method for the calculation of metabolic water. J. Physiol. 122, 399–402. 10.1113/jphysiol.1953.sp005009, PMID: 13118549 PMC1366125

[ref45] Navarro R. A. Meijer H. A. J. Underhill L. G. Mullers R. H. E. (2018). Extreme water efficiency of cape gannet Morus capensis chicks as an adaptation to water scarcity and heat stress in the breeding colony. Mar. Freshw. Behav. Physiol. 51, 30–43. 10.1080/10236244.2018.1442176

[ref46] Ostrowski S. Williams J. B. Bedin E. Ismail K. (2002). Water influx and food consumption of free-living oryxes (Oryx leucoryx) in the arabian desert in summer. J. Mammal. 83, 665–673. 10.1644/1545-1542(2002)083<0665:WIAFCO>2.0.CO;2

[ref47] Passey B. H. Ji H. (2019). Triple oxygen isotope signatures of evaporation in lake waters and carbonates: a case study from the western United States. Earth Planet. Sci. Lett. 518, 1–12. 10.1016/j.epsl.2019.04.026

[ref48] Passey B. H. Levin N. E. (2021). Triple oxygen isotopes in meteoric waters, carbonates, and biological apatites: implications for continental paleoclimate reconstruction. Rev. Mineral. Geochem. 86, 429–462. 10.2138/rmg.2021.86.13

[ref49] Pinheiro J. Bates D. DebRoy S. Sarkar D. Team R. C. (2013). nlme: Linear and nonlinear mixed effects models. *R Packag. version 3*, 111.

[ref50] R Development Core Team (2013). R: a language and environment for statistical computing.

[ref51] Remeš V. Harmáčková L. (2018). Disentangling direct and indirect effects of water availability, vegetation, and topography on avian diversity. Sci. Rep. 8:15475. 10.1038/s41598-018-33671-w, PMID: 30341321 PMC6195560

[ref52] Riddell E. A. Iknayan K. J. Hargrove L. Tremor S. Patton J. L. Ramirez R.. (2021). Exposure to climate change drives stability or collapse of desert mammal and bird communities. Science 371, 633–636. 10.1126/science.abd4605, PMID: 33542137

[ref53] Rutkowska J. Sadowska E. T. Cichoń M. Bauchinger U. (2016). Increased fat catabolism sustains water balance during fasting in zebra finches. J. Exp. Biol. 219, 2623–2628. 10.1242/jeb.138966, PMID: 27582561

[ref54] Sabat P. Cavieres G. Veloso C. Canals M. (2006a). Water and energy economy of an omnivorous bird: population differences in the rufous-collared sparrow (*Zonotrichia capensis*). Comp. Biochem. Physiol. - A Mol. Integr. Physiol. 144, 485–490. 10.1016/j.cbpa.2006.04.016, PMID: 16750645

[ref55] Sabat P. del Rio C. M. (2002). Inter- and intraspecific variation in the use of marine food resources by three Cinclodes (Furnariidae, Aves) species: carbon isotopes and osmoregulatory physiology. Zoology 105, 247–256. 10.1078/0944-2006-00078, PMID: 16351873

[ref56] Sabat P. Gonzalez-Vejares S. Maldonado K. (2009). Diet and habitat aridity affect osmoregulatory physiology: an intraspecific field study along environmental gradients in the rufous-collared sparrow. Comp. Biochem. Physiol. Part A Mol. Integr. Physiol. 152, 322–326. 10.1016/j.cbpa.2008.11.003, PMID: 19041952

[ref57] Sabat P. Maldonado K. Fariña J. M. Del Rio C. M. (2006b). Osmoregulatory capacity and the ability to use marine food sources in two coastal songbirds (Cinclodes: Furnariidae) along a latitudinal gradient. Oecologia 148, 250–257. 10.1007/s00442-006-0377-4, PMID: 16496181

[ref58] Schmidt-Nielsen K. (1997). Animal Physiology: Adaptation and Environment. New York: Cambridge University Press.

[ref59] Schoenemann S. W. Schauer A. J. Steig E. J. (2013). Measurement of SLAP2 and GISP δ17O and proposed VSMOW-SLAP normalization for δ17O and 17Oexcess. Rapid Commun. Mass Spectrom. 27, 582–590. 10.1002/rcm.6486, PMID: 23413217

[ref60] Şekercioğlu Ç. H. Primack R. B. Wormworth J. (2012). The effects of climate change on tropical birds. Biol. Conserv. 148, 1–18. 10.1016/j.biocon.2011.10.019

[ref61] Sharp Z. D. Gibbons J. A. Maltsev O. Atudorei V. Pack A. Sengupta S.. (2016). A calibration of the triple oxygen isotope fractionation in the SiO2–H2O system and applications to natural samples. Geochim. Cosmochim. Acta 186, 105–119. 10.1016/j.gca.2016.04.047

[ref62] Sharp Z. D. Wostbrock J. A. G. Pack A. (2018). Mass-dependent triple oxygen isotope variations in terrestrial materials. Geochem. Perspect. Lett 7, 27–31. 10.7185/geochemlet.1815

[ref63] Smit B. Mckechnie A. E. (2015). Water and energy fluxes during summer in an arid-zone passerine bird. Ibis 157, 774–786. 10.1111/ibi.12284

[ref64] Smit B. Woodborne S. Wolf B. O. McKechnie A. E. (2019). Differences in the use of surface water resources by desert birds are revealed using isotopic tracers. Auk Ornithol. Adv. 136:uky005. 10.1093/auk/uky005

[ref65] Smith E. K. O’Neill J. J. Gerson A. R. McKechnie A. E. Wolf B. O. (2017). Avian thermoregulation in the heat: resting metabolism, evaporative cooling and heat tolerance in Sonoran Desert songbirds. J. Exp. Biol. 220, 3290–3300. 10.1242/jeb.161141, PMID: 28684465

[ref66] Speakman J. Racey P. A. (1987). The equilibrium concentration of oxygen-18 in body water: implications for the accuracy of the doubly-labelled water technique and a potential new method of measuring RQ in free-living animals. J. Theor. Biol. 127, 79–95. 10.1016/S0022-5193(87)80162-5

[ref67] Tapia-Monsalve R. Newsome S. D. Sanchez-Hernandez J. C. Bozinovic F. Nespolo R. Sabat P. (2018). Terrestrial birds in coastal environments: metabolic rate and oxidative status varies with the use of marine resources. Oecologia 188, 65–73. 10.1007/s00442-018-4181-8, PMID: 29948312

[ref68] Tieleman B. I. Williams J. B. Buschur M. E. (2002). Physiological adjustments to arid and Mesic environments in larks (Alaudidae). Physiol. Biochem. Zool. 75, 305–313. 10.1086/341998, PMID: 12177833

[ref69] Vale C. G. Brito J. C. (2015). Desert-adapted species are vulnerable to climate change: insights from the warmest region on earth. Global Ecol. Conserv. 4, 369–379. 10.1016/j.gecco.2015.07.012

[ref70] Walther G.-R. Post E. Convey P. Menzel A. Parmesan C. Beebee T. J. C.. (2002). Ecological responses to recent climate change. Nature 416, 389–395. 10.1038/416389a, PMID: 11919621

[ref71] Whiteman J. P. Sharp Z. D. Gerson A. R. Newsome S. D. (2019). Relating Δ17O values of animal body water to exogenous water inputs and metabolism. Bioscience 69, 658–668. 10.1093/biosci/biz055

[ref72] Williams J. B. (2001). Energy expenditure and water flux of free-living dune larks in the Namib: a test of the reallocation hypothesis on a desert bird. Funct. Ecol. 15, 175–185. 10.1046/j.1365-2435.2001.00512.x

[ref73] Williams J. B. Siegfried W. R. Milton S. J. Adams N. J. Dean W. R. J. du Plessis M. A.. (1993). Field metabolism, water requirements, and foraging behavior of wild ostriches in the Namib. Ecology 74, 390–404. 10.2307/1939301

[ref74] Williams J. B. Tieleman B. I. (2000). Flexibility in basal metabolic rate and evaporative water loss among hoopoe larks exposed to different environmental temperatures. J. Exp. Biol. 203, 3153–3159. PMID: 11003826 10.1242/jeb.203.20.3153

[ref75] Williams J. B. Tieleman B. I. (2005). Physiological adaptation in desert birds. Bioscience 55, 416–425. 10.1641/0006-3568(2005)055[0416:PAIDB]2.0.CO;2

[ref76] Wostbrock J. A. G. Cano E. J. Sharp Z. D. (2020). An internally consistent triple oxygen isotope calibration of standards for silicates, carbonates and air relative to VSMOW2 and SLAP2. Chem. Geol. 533:119432. 10.1016/j.chemgeo.2019.119432

[ref77] Zuur A. Ieno E. N. Walker N. Saveliev A. A. Smith G. M. (2009). Mixed Effects Models and Extensions in Ecology with R New York: Springer Science and Business Media.

