# Supplementary material for: Triple Oxygen Isotope Measurements (Δ'17O) of Body Water Reflect Water Intake, Metabolism, and δ18O of Ingested Water in Passerines
Source: Front Physiol. 2021 Sep 6;12:710026. doi: 10.3389/fphys.2021.710026 (PMC8450417; doi:10.3389/fphys.2021.710026)
Supplement: Supplementary file 1 [file Table_1.DOCX]

Table S1. Individual Oxygen isotope values of body water and the estimated d18O of ingested water collected from acclimated *Zonotrichia capensis* in the lab and for *Cinclodes* species collected at the field.

| ***Z. capensis*** | Cold | | | | Warm | | | |
| --- | --- | --- | --- | --- | --- | --- | --- | --- |
| Individual | δ' ^17^O | | δ' ^18^O | Drink δ ^18^O | δ' ^17^O | δ' ^18^O | Drink δ ^18^O | |
| 3 | -3.23 | | -5.91 | -15.40 | -3.80 | -7.04 | -15.19 | |
| 11 | -1.15 | | -2.02 | -8.64 | 0.42 | 0.92 | -3.83 | |
| 8 | -2.24 | | -4.05 | -12.71 | -1.01 | -1.78 | -7.50 | |
| 10 | -2.92 | | -5.33 | -14.71 | -1.77 | -3.16 | -11.24 | |
| 12 | -1.16 | | -1.97 | -11.41 | -1.12 | -1.91 | -10.61 | |
| Mean±SD | -2.14±0.97 | | -3.86±1.83 | -12.67±2.71 | -1.46±1.53 | -2.59±2.90 | -9.67±4.26 | |
| ***C. nigrofumosus*** | |  | | | | | |  |
|  | | δ' ^17^O | | δ' ^18^O | Drink δ ^18^O | | | |
|  | | 5.02 | | 9.71 | 6.12 | | | |
|  | | -0.43 | | -0.679 | -5.70 | | | |
| Mean±SD | | 2.295±3.85 | | 4.52±7.35 | 0.21±8.32 | | | |
| ***C. oustaleti*** | |  | | | | | | |
|  | | 2.07 | | 4.152 | -2.65 | | | |
|  | | 2.88 | | 5.68 | -0.68 | | | |
|  | | 2.39 | | 4.72 | -0.95 | | | |
|  | | 1.69 | | 3.36 | -1.32 | | | |
| Mean ±SD | | 2.26±0.51 | | 4.48±0.98 | -1.40 ±0.87 | | | |
